# King's College Hospital

**Published:** 1905-06-17

**Authors:** 


### KING'S COLLEGE HOSPITAL.

### THE SELECTION OF PLANS.

When it was decided to remove King's College Hospital from its present site at the back of Lincoln's Inn Fields to Camberwell, the joint "Removal Committee" decided that a limited number of architects should be invited to submit plans in competition for the new building. Mr. Rowland Plumbe was appointed to assist the committee as assessor, and in conjunction with the medical staff of the rebuilding sub-committee drew up the instructions to competing architects. The latter were—W. A. Pite; Campbell, Douglas, and Paterson (Glasgow); E. T. Hall; A. Saxon Snell; W. Harvey; and Woodd and Ainslie.

The decision of the assessor was published a week or two back, and the plans have been on view at Carpenters' Hall during the past week. Mr. Plumbe's award places Mr. Pite's plan first, Messrs. Campbell, Douglas, and Paterson second, Mr. Snell third, and the remainder in the following order, Hall, Harvey, and Woodd and Ainslie.

A careful examination of the designs fully justifies the assessor's award, though it must be admitted that the design placed second runs the first very hard.

A main point in Mr. Pite's plan is simplicity of arrangement, all his large ward blocks are placed on one side of the main corridor and with their long axes north and south. Thus he not only gets the greatest possible access of sunlight and air, but also obtains at the south end of each pavilion a large balcony with an extensive prospect over the railway and the new public park.

It would be easy to criticise in detail Mr. Pite's plan as well as for the matter of that all the rest of the plans; but it must be admitted that the competitors had an almost impossible task set them in trying to plan the whole of the vast accommodation required on an inadequate site. Mr. Pite has produced a plan which in our judgment has only one serious competitor in Messrs. Douglas and Paterson; but if the work is to be carried through the whole scheme ought to be entirely remodelled, and either the accommodation reduced or more land acquired. Probably the latter alternative is the right one; and we believe the question of acquiring a separate site for the out-patient department has already been before the Removal Committee.

With the out-patient department removed to another site the administration block could be remodelled and the narrow interior courts, which could never receive sufficient light and air, could be either omitted altogether or so remodelled as to be unobjectionable; more suitable sites could be found for the students' hostel and the houses for the medical superintendent and secretary than the edge of the slope of a deep railway cutting, and the buildings generally could be improved by greater facilities for circulation of air and light.

